# Diabetes Susceptibility Assessment Using the Indian Diabetes Risk Score: A Cross-Sectional Analytical Study on Young Medical Students in Chennai, South India

**DOI:** 10.7759/cureus.49795

**Published:** 2023-12-01

**Authors:** Rushender Rajan, Logaraj Muthunarayanan

**Affiliations:** 1 Preventive and Social Medicine, St. Peter’s Medical College, Hospital and Research Institute, Hosur, IND; 2 Community Medicine, SRM Medical College Hospital and Research Centre, SRM Institute of Science and Technology, Chengalpattu, IND

**Keywords:** undergraduate medical students, diabetes mellitus, india, risk assessment tools, who-steps

## Abstract

Background

Youth must identify their diabetes risk factors because the disease is currently affecting young people at an epidemic rate. The Indian Diabetes Risk Score (IDRS) is a reliable and affordable method for determining a person’s risk of developing diabetes. This study aims to evaluate the ability of the IDRS to predict type 2 diabetes mellitus (T2DM) and investigate the relationship between diabetes risk and other variables in young medical students.

Methodology

In this cross-sectional analytical survey of a cohort of 670 undergraduate medical scholars over six months, a meticulously designed semi-structured questionnaire was administered. The questionnaire captured sociodemographic specifics, substance use habits (tobacco and alcohol), dietary patterns, and physical activity patterns in line with the WHO STEPwise approach to non-communicable disease risk factor surveillance and the IDRS tool.

Results

There was an equal proportion of males and females. The majority were in their second year of study. Overall, 5.2% of students reported tobacco use, while alcohol consumption was reported by 25.6% of the cohort. Furthermore, IDRS score insights revealed that 80.2% of the participants exhibited low diabetes risk, 18.9% were categorized as moderate risk, and 0.9% demonstrated high risk. Gender (adjusted prevalence ratio (PR) = 1.8, 95% confidence interval (CI) 1.3-2.7) and tobacco usage (adjusted PR = 1.6 (1.1 to 2.2)) were found to be significantly associated with higher IDRS scores on multivariable analysis.

Conclusions

Illuminating the landscape of risk determinants among medical students in Chennai, this study accentuates the pressing need for bespoke interventions, underpinned by behavioral change theories. Such strategies can potentially catalyze healthier lifestyle shifts, shaping favorable public health trajectories in the foreseeable future.

## Introduction

Diabetes mellitus is a predominant factor in global mortality and morbidity, putting significant strain on public health systems worldwide. While the condition surfaces during adulthood, the foundation often lies in early life choices and habits. The World Health Organization has reported that non-communicable diseases (NCDs) account for about 31% of all global deaths, with a concerning 75% of deaths occurring in low- and middle-income countries, with diabetes being the major cause next to cardiovascular diseases [1]. A majority of these deaths can be prevented as they are primarily linked to modifiable risk factors such as tobacco use, an unhealthy diet, lack of physical activity, and excessive alcohol consumption [2]. Moreover, according to data, South Asians are diagnosed with diabetes nearly a decade earlier than individuals in Western countries [3].

Addressing these risk factors early on is pivotal, both for individual health and larger public health objectives. This underpins the importance of risk assessment, particularly to instigate lifestyle changes in the younger generation who might be at potential risk [4]. To craft effective interventions, there is a need to fully understand and address the prevalence of these risk factors among young adults. However, there is a major gap in awareness; many young individuals are unaware of the risks, leading to a potential underestimation of the problem [5].

The Madras Diabetes Research Foundation created and developed the Indian Diabetes Risk Score (IDRS), a validated instrument to identify people at high risk of acquiring type 2 diabetes mellitus (T2DM) in the future. It takes into account the following four risk factors: physical activity, age, family history, and abdominal obesity [6]. Due to less attention being paid to medical students, little research has been done on diabetes screening in this population.

Medical students, despite their role as future healthcare advocates, are not exempt from these risk factors. Their rigorous academic demands, unpredictable eating patterns, sedentary lifestyles, and stress can often lead them down a path of unhealthy habits. Their unique academic environment and lifestyle can indeed make them more susceptible to these risks [7]. Existing research has highlighted the prevalence of risk factors among healthcare students [8-11]. There is a pressing need to enhance awareness and knowledge about these risk factors among medical students [7]. While some studies have focused on the prevalence of these factors among medical students in India, there is a noticeable gap, especially regarding data from the eastern part of the country [9-11]. With this in mind, the goal of the current study, which involved undergraduate medical students, was to evaluate the ability of the IDRS to predict T2DM and investigate the relationship between diabetes risk and other sociodemographic factors.

## Materials and methods

Study design, settings, and period

We performed a cross-sectional analytical study among medical students at a tertiary care center in Chennai, South India. Chennai is the capital city of the Tamil Nadu state and boasts a rich heritage of renowned medical institutions. Our specific focus was on a private university that predominantly serves the communities located on the city’s outskirts. From the university’s line list, we recruited medical students through simple random selection. The data collection spanned over six months from December 2021 to May 2022. The primary motivation behind this study was to serve as an initial survey for a more comprehensive research project. This larger study aimed to determine the efficacy of mobile-based lifestyle interventions in ameliorating obesity among university students in Chennai. This study was approved by the Institutional Ethics Committee of SRM Medical College (approval number: 1665/IEC/2019).

Study participants

Our participant pool encompassed medical college undergraduate students registered with the chosen university. To streamline our approach, we drafted a list of undergraduate students using university registers. Using this list as our sampling frame, we employed simple random sampling to select study participants. The questionnaire, specifically tailored to assess lifestyle habits, was dispensed during regular theory and practical classes. It is worth noting that students who remained absent for three days were not included in the study.

Sample size

The sample size was calculated by incorporating the findings of the study by Chakma et al [12]. In their findings, 28.66% of college students exhibited behaviors, and factoring in an 80% power with a two-sided alpha error of 5%, we determined that a sample size of 670 would be ideal. Our calculations for sample size were done through OpenEpi Version 3.01.

Study procedure

After securing approval from the Institute Ethics Committee, the study commenced. Each participant was informed about the study’s objectives, after which written informed consent was obtained, ensuring participants’ voluntary involvement.

To facilitate data collection, we used a pretested, semi-structured questionnaire designed to capture sociodemographic details and behavioral risk factors associated with diabetes. The foundation for our questionnaire was the WHO STEPwise approach to NCD risk (STEPS) factor surveillance instrument and IDRS, renowned for its comprehensive approach to assessing cardiovascular disease risk factors and predicting diabetes [13]. Experts from the Department of General Medicine meticulously reviewed and refined the questionnaire, ensuring its relevance and clarity. Operational definitions are presented in Table 1.

**Table 1:** Operational Definitions

Term	Definition
Tobacco use	Any consumption of tobacco products, whether smoking or smokeless, within the past 30 days
Alcohol consumption	Any intake of alcoholic beverages in the past 30 days
Physical activity	Evaluated based on frequency, duration, and intensity of both work-related and recreational physical activities. Levels are classified as low, moderate, or high
Dietary habits	Assessed based on the average intake of fruits, vegetables, and other dietary patterns over the past seven days
Waist circumference	Measured using a non-stretchable measuring tape, with the participant in a standing position, midway between the lower rib and the iliac crest
Height	Measured using a stadiometer with participants standing upright without shoes
Weight	Assessed with a calibrated digital scale, with participants wearing minimal clothing
Body mass index	Computed by dividing weight (in kilograms) by the square of height (in meters). Categories include underweight, normal weight, overweight, and obese

Indian Diabetes Risk Score

IDRS was developed using four simple parameters, namely, age, abdominal obesity, family history of diabetes, and physical activity. Subjects with an IDRS <30 were categorized as low risk, 30-50 as medium risk, and those with >60 as high risk for diabetes [14].

After the questionnaire’s formulation, questions such as year of study, place of stay, and anthropometric measurements such as waist circumference, were digitalized into Google Forms. Data collection employed an interview technique, where pertinent questions were discussed with participants to gather comprehensive insights.

For a systematic approach to data gathering from MBBS students across various years, we identified a representative from each batch. These representatives were entrusted with sharing the Google Forms link among their peers via WhatsApp and email, ensuring optimal reach. To uphold the privacy of the participants, their names and any personally identifiable information were kept anonymous throughout the study process.

Data analysis

The responses collected through Google Forms were diligently exported to Microsoft Excel (Microsoft Corp., Redmond, WA, USA) for preliminary data organization. Subsequently, the data were imported into the SPSS software version 25 (IBM Corp., Armonk, NY, USA) for thorough analysis. Continuous numerical variables, such as age and anthropometric measurements, were articulated as mean ± standard deviation. Categorical variables, representing specific risk factors and behavioral patterns, were depicted in terms of frequency (number of occurrences) and proportions (percentage of the total sample).

Using log-binomial regression, the relationship between the IDRS risk score and each of the independent variables, such as gender, year of study, tobacco use, alcohol use, outside food consumption, and weekly fruit intake, was evaluated. Results were presented as a prevalence ratio (PR) with a 95% confidence interval (CI). For multivariate logistic regression, only the variables that showed statistical significance at a p-value <0.2 in log-binomial regression were taken into account. P-values <0.05 were considered statistically significant in the adjusted analysis.

## Results

The mean age of the participants was 19.9 (±1.5) years. The gender distribution reflected that 50.4% of the participants were female. The sociodemographic characteristics revealed that 173 (26%) students hailed from a rural background, while 181(27%) were enrolled in their second year of medical education. Regarding parental education, 578 (86%) students’ fathers and 578 (62%) mothers had attained at least secondary education or higher. Furthermore, 649 (97%) fathers and 415 (77%) mothers were employed (Table 2).

**Table 2:** Sociodemographic characteristics of the study participants (N = 670).

Characteristics	Frequency (%)
Mean age of the participants = 19.4	
Gender	
Male	332 (49.5)
Female	338 (50.5)
Year of study	
1st year	171 (25.5)
2nd year	181 (27.1)
3rd year	175 (26.1)
4th year	143 (21.3)
Father’s education	
Illiterate	17 (2.5)
Primary education	75 (11.1)
Secondary education and higher	578 (86.2)
Mother’s education	
Illiterate	58 (8.6)
Primary education	193 (28.8)
Secondary education and higher	578 (62.6)
Father’s employment	
Unemployed	21 (3.1)
Employed	649 (96.9)
Mother’s employment	
Unemployed	152 (22.7)
Employed	415 (77.3)
Residence	
Urban	497 (74.2)
Rural	173 (25.8)

Tobacco and alcohol consumption patterns

Our data (presented in Table 3) indicated that 35 (5.2%) students were current users of tobacco or related products. Notably, about 13 (2%) respondents consumed tobacco daily. The mean age at which these students initiated tobacco use was 20.5 years. When evaluating the use of smokeless tobacco, the frequency stood at 8 (1%), with a daily consumption rate of 0.6%. In terms of alcohol consumption, 172 (25.6%) acknowledged having consumed alcohol at some point in their lives. More recent consumption trends showed that 93 (14%) had consumed alcohol in the past 30 days, and 159 (24%) had consumed within the previous 12 months.

**Table 3:** Distribution of tobacco and alcohol usage and dietary habits among study participants (N = 670).

Nutrition and addiction habits	Frequency (%)
Do you currently smoke any tobacco products, such as cigarettes, cigars, or pipes?	
Yes	35 (5.2)
No	635 (94.7)
Do you smoke daily?	
Yes	13 (1.9)
No	657 (98.1)
How old were you when you first started smoking daily?	
Mean (SD)	20.5 (1.6)
Do you currently use any smokeless tobacco such as (snuff, chewing, tobacco, betel)?	
Yes	8 (1.1)
No	5 (98.9)
Do you currently use smokeless tobacco products daily?	
Yes	4 (0.6)
No	666 (99.4)
Have you ever consumed an alcoholic drink?	
Yes	172 (25.6)
No	494 (73.7)
Have you consumed an alcoholic drink within the past 30 days?	
Yes	93 (13.8)
No	577 (86.2)
Have you consumed an alcoholic drink within the past 12 months?	
Yes	159 (23.7)
No	511 (76.3)
In a typical week, on how many days do you eat fruit?	
Nil	81 (12.1)
1–2 days	229 (34.2)
3–6 days	235 (35.1)
>6 days	125 (18.6)
How many servings of fruit do you eat on one of those days?	
Nil	81 (12.1)
1–2 servings	548 (81.8)
3–4 servings	41 (6.1)
In a typical week, on how many days do you eat vegetables?	
Nil	47 (7.0)
1–2 days	429 (64.0)
3–4 days	138 (20.5)
5–6 days	51 (7.6)
>7 days	5 (0.7)
How many servings of vegetables do you eat one of those days?	
Nil	47 (7.0)
1–2 servings	581 (86.7)
3–4 servings	42 (6.2)
Do you eat outside food other than home food?	
Yes	573 (85.5)
No	97 (14.5)
On average, how many meals per week do you eat outside other than home or hostel food?	
Nil	97 (14.5)
1–2 days	477 (71.3)
3–4 days	60 (8.9)
5–6 days	28 (4.1)
>7 days	8 (1.2)

Dietary habits

As detailed in Table 3, 589 (88%) medical students consumed fruits at least once daily, with a typical serving comprising one to two servings of fruits such as bananas, pineapples, watermelons, and papayas. Regarding vegetable consumption, an impressive 623 (91%) respondents included vegetables in their daily diet. Additionally, 573 (85.5%) students reported consuming meals outside their home or hostel at least once.

Physical activity patterns

The data on physical activity patterns highlighted that a mere 117 (17%) students were involved in vigorous-intensity sports activities such as running or football. On the other hand, 244 (36%) engaged in moderate-intensity sports activities. A noteworthy 85 (12%) respondents reported walking or cycling.

Indian Diabetes Risk Score

An assessment of the IDRS scores of the participants provided critical insights into diabetes susceptibility among the cohort. Our findings indicated that the majority, i.e., 537 (80.2%) of the students, fell into the category of low diabetes risk. In contrast, 127 (18.9%) demonstrated moderate risk, and a concerning six (0.9%) were identified as being at high risk for diabetes (Table 4).

**Table 4:** Distribution of components of IDRS among study participants (N = 670). IDRS = Indian Diabetes Risk Score

IDRS components		Frequency (percentage)
Waist circumference (cm)	<80 Female	271 (40.3)
	<90 Male	238 (35.5)
	>81–89 Female	47 (7.1)
	>91–99 Male	47 (7.1)
	>90 female	20 (2.9)
	>100 male	47 (7.1)
Physical activity	Vigorous exercise	117 (17.5)
	Moderate exercise	244 (36.4)
	Mild exercise	85 (12.7)
	No exercise	224 (33.4)
Family history of diabetics	Both parents are non-diabetic	531 (79.3)
	One parent is diabetic	116 (17.3)
	Both parents are diabetic	23 (3.4)
IDRS score	Low	537 (80.2%)
	Moderate	127 (18.9%)
	High	6 (0.9%)

For regression analysis, IDRS was categorized as low and moderate to high risk. On unadjusted analysis with log-binomial regression, gender, year of study, tobacco usage, alcohol consumption, and place of study were found to be significant with p-values <0.2. On further adjusted analysis, males had an increased proportion (adjusted PR = 1.8, 95% CI = 1.3 to 2.7) of higher IDRS than females, and tobacco usage had an increased proportion (adjusted PR 1.6 (1.1 to 2.2)) of higher IDRS than non-tobacco users, and both were statistically associated factors with p-value of <0.05 (Table 5).

**Table 5:** Unadjusted and adjusted analysis of selected factors with IDRS among the study participants (N = 670). IDRS = Indian Diabetes Risk Score; PR = prevalence ratio; CI = confidence interval *: Unadjusted p-value <0.2; #: Adjusted p-value <0.05.

Characteristics		IDRS risk				Unadjusted PR (95% CI)	Adjusted PR (95% CI)	P-value
		Low (N = 537)		Moderate to high (N = 133)				
		N	%	N	%			
Gender	Female	287	84.9%	51	15.1%	Reference	Reference	<0.001^#^
	Male	250	75.3%	82	24.7%	1.8 (1.3 to 2.7)*	1.8 (1.3 to 2.7)	
Year of study	1st year	138	80.7%	33	19.3%	Reference	Reference	
	2nd year	156	86.1%	25	13.9%	0.7 (0.4 to 1.1)*	0.6 (0.3 to 1.1)	0.19
	3rd year	131	74.8%	44	25.2%	1.3 (0.9 to 1.9)*	1.2 (0.7 to 1.9)	0.08
	4th year	112	78.3%	31	21.7%	1.1 (0.7 to 1.7)	0.9 (0.5 to 1.4)	0.70
Smoking status	No	512	80.6%	123	20.4%	Reference	Reference	0.04^#^
	Yes	25	71.4%	10	28.5%	1.8 (1.1 to 2.3)*	1.6 (1.1 to 2.2)	
Alcoholic status	No	392	78.7%	106	21.3%	1.7 (0.9 to 3.1)*	1.3 (0.9 to 2.7)	0.10
	Yes	145	84.3%	27	15.7%	Reference	Reference	
Outside food consumption	No	73	13.1%	24	18.1%	Reference	-	-
	yes	464	86.5%	109	81.9%	0.7 (0.5 to 1.1)		
Place of staying	DS	406	81.7%	91	18.3%	Reference	Reference	0.06
	Hostel	131	75.2%	42	24.8%	1.4 (0.9 to 1.9)*	1.3 (0.9 to 1.8)	
Fruit consumption	0 times	66	81.3%	15	19.7%	Reference	-	0.733
	1–3 times in a week	182	79.5%	47	20.5%	0.5 (0.1 to 1.5)		
	3–6 times in a week	197	83.8%	38	16.2%	0.4 (0.1 to 1.3)		
	>6 times in a week	92	73.6%	33	26.4%	0.7 (0.2 to 2.1)		

## Discussion

NCDs are witnessing an increasing trend among the working-age population of developing nations. Understanding the burden of diabetic risk factors in adolescents and young adults is vital, especially because obesity rates are rising within this demographic. A significant proportion of these risk factors, encompassing dietary habits, tobacco and alcohol consumption, and physical activity, are preventable and modifiable. In our study, involving 670 medical undergraduate students, we utilized the WHO STEPs questionnaire to assess the prevalence of certain lifestyle disease risk factors. The results revealed that 5.2% of students smoked or used tobacco products, 25.6% consumed alcohol, 88% and 91% consumed fruits and vegetables daily, respectively, and 17% engaged in vigorous physical activity.

Comparatively, our study findings present intriguing contrasts and similarities with global data. For instance, the tobacco consumption rate in our study was 5.2%, with an average initiation age of 19, echoing the estimates from the Global Adult Tobacco Survey. This rate was notably lower than findings from studies in Saudi Arabia, Bangladesh, and Nepal [14-16]. Conversely, the alcohol consumption rate of 25.6% matched the data from other Indian studies [17]. One plausible explanation for the lower tobacco usage could be the heightened awareness of the long-term detrimental effects of smoking, rigorous anti-smoking campaigns, stringent tobacco control measures, and cultural attitudes toward smoking. Our findings related to tobacco use parallel several other Indian studies, underscoring the regional nuances of tobacco consumption [17,18].

Our data highlighted that 88% of the students consumed fruits at least once daily, with nearly 88% consuming one to two servings, and 91% reported daily vegetable consumption. These findings are in line with prior research conducted in India [19,20].

Notably, our results showed that a sizable percentage of the students, i.e., 80.1%, had a low diabetes risk. This finding is consistent with past epidemiological research showing that a sizable proportion of the Indian population had a low risk of developing diabetes [21]. This observation is in line with other research that suggests medical students have lower diabetes risk factors than the general population because they are younger and possibly more health conscious [22]. In contrast, 18.9% of the individuals exhibited a moderate risk for diabetes, indicating an increased necessity for proactive preventive interventions, including regular health screenings and lifestyle adjustments. Furthermore, the identification of 0.9% of the cohort at high risk for diabetes highlights the importance of early risk assessment, especially among individuals who may be susceptible due to genetic or lifestyle factors.

The findings of the multivariable analysis are consistent with earlier research that showed gender discrepancies in metabolic health and emphasizes the significance of taking gender into account when measuring IDRS [23]. Furthermore, another study conducted among undergraduate students from north India also supported the fact that males are at a higher risk [24]. Studies also support the documented link between tobacco use and harmful metabolic consequences at a very early age [25,26]. With p-values of less than 0.05 in the adjusted analysis, our findings, which are based on meticulous adjusted analyses, strongly support the claim that gender and tobacco use are independent predictors of elevated IDRS, highlighting their clinical relevance and significance for upcoming preventive and therapeutic initiatives.

A health-promoting lifestyle is a pivotal determinant of health status, serving as a cornerstone for health sustenance and enhancement. The observed prevalence of tobacco and alcohol usage among medical students can be dissected using various social and behavioral frameworks. The Theory of Planned Behaviour suggests that behaviors stem from intentions, molded by individual attitudes, societal norms, and perceived behavioral control [27]. The influence of peer behavior and societal perceptions can considerably steer young adults toward or away from tobacco and alcohol. Additional stressors such as academic challenges, peer pressure, and limited recreational options might push students toward smoking and drinking as coping strategies. This perspective aligns with the Self-Medication Hypothesis, implying self-regulated substance use to counteract stress and emotional distress. On a brighter note, the high consumption rates of fruits and vegetables in our sample might find its roots in the Health Belief Model. This model posits that individuals would adopt health-promoting behaviors if they perceive disease threats and understand the behavior’s benefits. Such positive dietary habits might be fostered by effective health campaigns and consistent health education. Therefore, youth should be a key stakeholder in the policy-making process and not merely a policy target [28].

Strengths and limitations

Our study is one among the very few that have investigated the burden of diabetes risk factors among medical students. The use of the WHO STEPS questionnaire enhances the study’s reliability and comparability with global data. The large sample size and simple random selection of students contribute to the generalizability of findings as most medical students are exposed to similar characteristics. However, the study has some limitations. First, the self-reported nature of data on tobacco, alcohol, and dietary habits might be influenced by recall and social desirability bias. Second, the cross-sectional design limits our ability to infer causality but we have used PR instead of odds ratio to better justify the association.

## Conclusions

Our study sheds light on the prevalence of diabetes risk factors among medical students in a South Indian university. The observed prevalence of tobacco and alcohol use calls for urgent attention from policymakers, institutions, and healthcare providers. Targeted interventions based on behavior change theories can empower these future healthcare professionals to adopt healthier lifestyles, setting an example for their patients and positively impacting public health outcomes. Educational initiatives must focus on altering attitudes, norms, perceptions, and perceived control related to tobacco and alcohol consumption. These interventions should be culturally sensitive and should consider the social determinants of health within the region.

Youth are change enablers and the future leaders of the world. With NCD being a human rights issue, governments must take cognizance of the problem’s magnitude, especially in low- and middle-income countries. In India, the current youth cohort will be living proof of the effectiveness of NCD prevention policies in curbing rising trends of chronic diseases. Therefore, youth should be a key stakeholder in the policy-making process and not merely a policy target.
